# Editorial: Digital technology for oral health care and dental education

**DOI:** 10.3389/fdmed.2025.1641791

**Published:** 2025-06-18

**Authors:** Kunaal Dhingra, Prabhat Kumar Chaudhari, Jitendra Sharan, Anand Marya, Dian Jing

**Affiliations:** ^1^ Division of Periodontics, Centre for Dental Education and Research, All India Institute of Medical Sciences, New Delhi, India; ^2^ Division of Orthodontics, Centre for Dental Education and Research, All India Institute of Medical Sciences, New Delhi, India; ^3^ Department of Dentistry, All India Institute of Medical Sciences Bhubaneswar, Odisha, India; ^4^ Faculty of Dentistry, University of Puthisastra, Phnom Penh, Cambodia; ^5^ Department of Orthodontics, China Shanghai Ninth People’s Hospital, College of Stomatology, Shanghai Jiao Tong University School of Medicine, Shanghai, China

**Keywords:** digital, oral health, dentistry, dental education, technology

**Editorial on the Research Topic**
Digital technology for oral health care and dental education

In recent years, digital technologies have rapidly transformed the landscape of oral health care and dental education, driving innovation, efficiency, and inclusivity across the globe. From artificial intelligence (AI) and intraoral scanning to virtual reality and tele-dentistry, these tools are not only enhancing the accuracy of diagnosis and treatment planning but also revolutionizing the way dental professionals are trained and how efficiently the oral care is delivered, particularly in underserved populations.

This special issue, titled “*Digital Technology for Oral Health Care and Dental Education*”, brings together a diverse set of scholarly contributions that explore the intersection of emerging digital technologies with various aspects of clinical practice, community outreach, and educational innovation. The four featured articles provide a unique window into the potential and challenges of integrating technology into the dental profession, each offering important insights into different dimensions of this evolving field.

The first article by Lubanga et al. explores how digital technologies and developmental accelerators can bridge persistent oral health disparities in Africa and contribute to achieving the Sustainable Development Goals (SDGs) and Africa's Agenda 2063. Despite oral diseases affecting nearly 44% of sub-Saharan Africa's population, the region suffers from severe underinvestment in oral healthcare, with most countries spending less than $1 per person annually. This underfunding, coupled with a shortage of trained personnel and inadequate infrastructure, limits access to quality oral health services, especially in rural and marginalized communities. The authors highlight the promise of digital tools such as mobile health (mHealth), telemedicine, electronic health records, and AI in overcoming systemic barriers. Case studies from other health domains demonstrate that SMS-based interventions and AI-assisted diagnostics can improve access, early detection, and treatment outcomes. These technologies can also support training of community health workers, remote consultations, and data-driven policymaking. The article argues that integrating digital innovations into oral healthcare systems is essential for equitable and efficient care. It calls for targeted investments, inclusive policies, and infrastructure improvements to expand digital reach, especially in underserved areas. Emphasizing contextual application, the authors advocate for a strategic and inclusive digital transformation of oral health systems in Africa.

The second article by Panaite et al. is a cross-sectional study that explored Romanian orthodontists' perceptions, practices, and challenges associated with use of orthodontic mini-implant, a temporary anchorage device. Conducted via a validated online survey with 159 specialists, results showed high recognition of mini-implant benefits—improved treatment efficiency, fewer side effects, and reduced patient compliance. Titanium implants were preferred, particularly for tasks like molar uprighting and anterior retraction. However, key barriers included lack of hands-on training, concerns about complications like soft-tissue irritation, and patient anxiety. Female and less experienced orthodontists often referred implant placements to maxillofacial surgeons due to limited confidence. Despite these challenges, over 80% of participants anticipated increased future use of mini-implants. Complications such as screw loosening and hygiene difficulties were common, but satisfaction with orthodontic treatment outcomes remained high. The study highlights the need for enhanced training programs to close knowledge gaps and improve implant success. While findings align with global research, limitations such as geographic focus and self-report biases suggest the need for broader studies.

The third article by Roselli et al. aimed to compare the reliability of digital impressions from intraoral scanners (IOS) as compared to alginate impression for recording palatal rugae (PR). Nineteen adult participants were enrolled, and PR images were obtained using alginate impressions, digital scans, and clinical photography. Six specific landmarks on each PR image were analyzed using FaceComp™ software, which measured distances and shape characteristics. Statistical analysis showed no significant difference between the digital and analog methods, both correlating strongly with clinical photographs. Results suggested that digital impressions are as accurate as traditional methods, offering advantages in speed, efficiency, and reproducibility. This has important implications for both clinical orthodontics and forensic dentistry, especially in edentulous or disfigured individuals. Limitations include small sample size and scanner variability. Nevertheless, the study supports the use of IOS for PR analysis and identification, paving the way for broader digital integration in dental and forensic practices.

The final contribution in this special issue is a descriptive case report by Ronsivalle et al. which details the multidisciplinary orthodontic management of a 50-year-old female patient with stage 3, grade B periodontitis and severe anterior mandibular crowding. Malocclusion and periodontal disease often coexist in adults, as malalignment can worsen plaque accumulation and occlusal trauma. Despite periodontitis not being a contraindication for orthodontics, special care must be taken due to the compromised alveolar bone. A two-phase treatment was implemented: initial non-surgical periodontal therapy (scaling and root planing) to stabilize inflammation, followed by orthodontic treatment using clear aligners. Clear aligners were chosen for their ability to maintain oral hygiene, deliver light forces, and enable precise biomechanical control. Interproximal reduction (IPR) and arch expansion created space without extractions. Treatment aimed to align the teeth, improve aesthetics, and preserve periodontal health. The patient showed excellent compliance and achieved all treatment goals in under a year, with stable occlusion, improved incisor inclination, better smile aesthetics, no signs of active periodontal disease, and improved quality of life. Clear aligners allowed for a customized, minimally invasive approach, particularly beneficial for patients with periodontal risk. The digital planning platform also enhanced patient understanding and satisfaction. Overall, this case highlights clear aligners as an effective and safe option in the orthodontic management of adult periodontal patients.

Collectively, the articles featured in this special issue illustrate the profound and varied ways in which digital technologies are reshaping the practice, science, and teaching of dentistry. From community-based oral health interventions in Africa to the precise control of clear aligners in patients with periodontal complications, these contributions highlight a central truth: digital tools are not simply augmenting traditional methods but they are redefining them.

As we look ahead, it is clear that digital dentistry is no longer a futuristic concept; it is today's imperative. And with continued research, cross-disciplinary collaboration, and a commitment to equity, we can ensure that digital technology becomes a powerful enabler of better, broader, and more personalized oral health care for all.

